# Unexpected Massive Hemorrhage During Incision and Drainage of a Presumed Perianal Abscess: A Case of Pelvic Chondrosarcoma

**DOI:** 10.7759/cureus.112035

**Published:** 2026-07-04

**Authors:** Rishi Meswani, Kajal Mitra, Prashant Onkar, Suresh Phatak, Anushika Maloo

**Affiliations:** 1 Radiodiagnosis, N. K. P. Salve Institute of Medical Sciences & Research Centre, Nagpur, IND; 2 Radiodiagnosis, Datta Meghe Institute of Higher Education & Research, Wardha, IND

**Keywords:** endosteal expansion, malignant bone tumor, malignant chondrosarcoma, ring and arc, soft tissue extension

## Abstract

Chondrosarcoma is a malignant cartilaginous neoplasm that commonly affects the pelvis. Due to its indolent growth pattern and deep anatomical location, pelvic disease may remain undetected until the lesion reaches a considerable size. In the present case, a 62-year-old male presented with a presumed perianal abscess and underwent incision and drainage, leading to unexpected massive hemorrhage intraoperatively. A subsequent contrast-enhanced CT scan demonstrated a large destructive pelvic lesion containing chondroid-type calcifications and prominent internal vascular channels. This case highlights the importance of radiological evaluation when clinical findings are atypical or inconclusive.

## Introduction

Chondrosarcoma is the third most common primary malignant tumor of bone, following myeloma and osteosarcoma [1]. Conventional forms of chondrosarcoma are generally slow-growing and may demonstrate favorable outcomes after adequate surgical resection [1]. The tumor most commonly affects adults between 30 and 70 years of age and shows a male predominance [2]. Clinically, patients usually complain of pain and tenderness, with or without an associated palpable swelling. Because pelvic tumors are deeply situated and often enlarge gradually, symptoms may persist for prolonged periods before diagnosis, frequently for one to two years [2]. Common sites of involvement include the pelvis, femur, and humerus [2].

Pathologically, these tumors characteristically form lobules of cartilage with variable central cystic or necrotic degeneration [2]. Resistance to chemotherapy and radiotherapy has been attributed to the abundant extracellular matrix, poor vascularity, and relatively low fraction of actively dividing cells within the tumor [1]. Consequently, wide surgical excision remains the preferred treatment for intermediate- and high-grade lesions [1,3,4].

Although a perianal abscess is usually diagnosed clinically, other conditions, including anorectal malignancies, soft tissue sarcomas, chronic hematomas, and pelvic tumors extending into the perineum, may occasionally mimic this presentation, particularly when clinical findings are atypical. The present case underlines the importance of imaging before intervention and the need to consider underlying malignancy when the clinical picture is not entirely typical.

## Case presentation

A 62-year-old male presented with pain and swelling involving the left perianal region. Clinical examination revealed a diffuse, tender swelling extending from the 4 to 6 o’clock position with an associated local rise in temperature. No discharge was identified, and a definite underlying mass could not be confidently appreciated clinically. Based on these findings, a diagnosis of perianal abscess was made, and incision and drainage were planned without prior imaging.

During the procedure, sudden, profuse hemorrhage was encountered from the operative site and was difficult to control. In addition, atypical tissue fragments encountered intraoperatively raised suspicion that this was not a routine abscess. The procedure was therefore terminated, the wound was packed, and the estimated blood loss was approximately 1.5 L (Figure 1). The patient was subsequently stabilized with transfusion support, after which an urgent CT angiographic study was performed to determine the source of bleeding.

**Figure 1:**
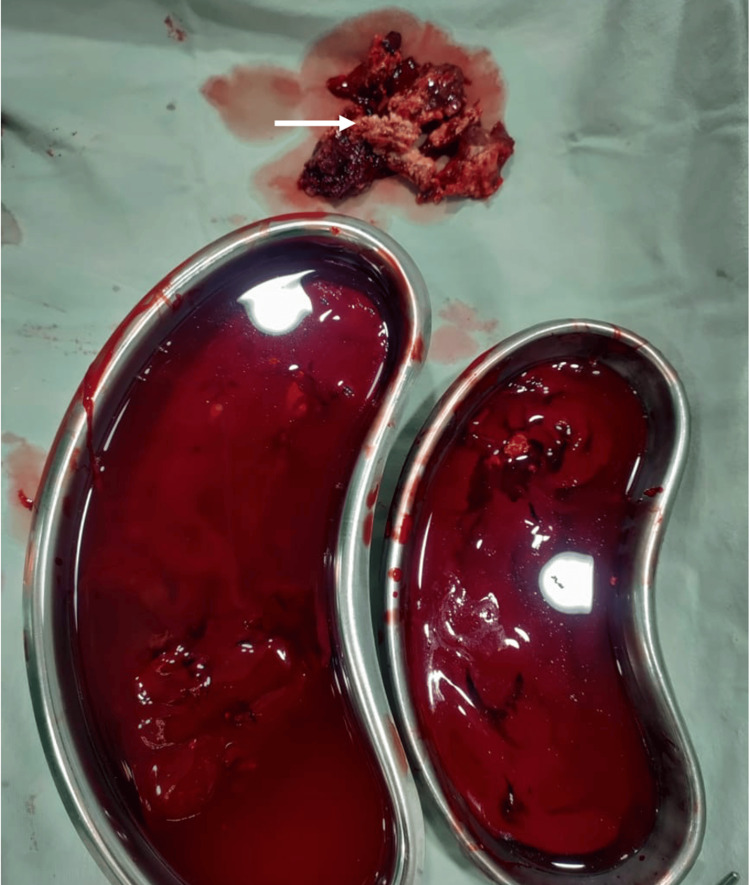
Intraoperative blood loss with retrieved tissue fragments Two kidney trays containing approximately 1.5 L of blood with suspicious tissue fragments (white arrow) were obtained during attempts to control the hemorrhage.

Contrast-enhanced CT of the pelvis revealed a large lobulated expansile lytic lesion involving the left pubis and ischium, measuring approximately 12.3 × 10.5 × 13.9 cm (anteroposterior × transverse × craniocaudal). The lesion demonstrated heterogeneous soft tissue attenuation with multiple internal ring-and-arc calcific densities consistent with a chondroid matrix. There was associated cortical destruction, along with a large extraosseous soft tissue component (Figure 2).

**Figure 2:**
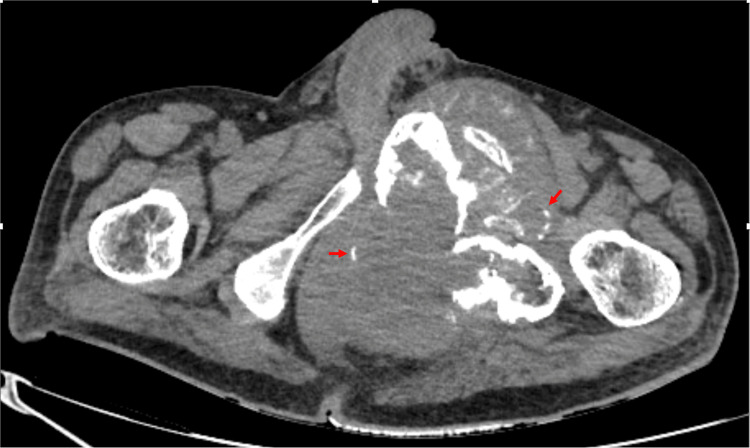
Non-contrast CT of the pelvis Non-contrast CT reveals a large expansile lesion involving the left pubic bone, causing cortical destruction and expansion, with multiple foci of stippled calcification in a ring-and-arc pattern (red arrow).

Post-contrast images showed peripheral enhancement with multiple irregular enhancing vascular channels traversing the lesion. Central non-enhancing areas likely represented cartilaginous and necrotic components (Figure 3). The vascular supply appeared to arise predominantly from branches of the left internal iliac artery, with abnormal vascular channels extending inferiorly toward the operative site to the surgical gauze used for intraoperative packing (Figure 4).

**Figure 3:**
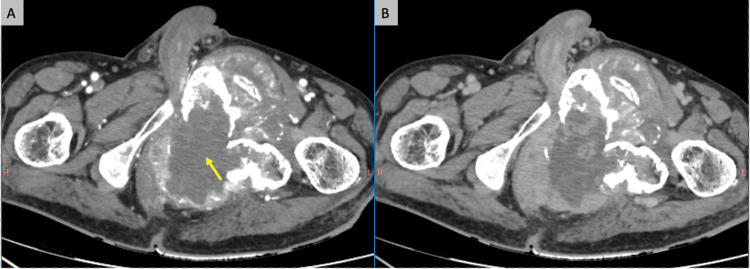
Contrast-enhanced CT of the pelvis in the arterial phase (A) and venous phase (B) The lesion showed significant peripheral vascularity with central non-enhancing fluid-attenuation areas (yellow arrow), likely representing the chondroid matrix.

**Figure 4:**
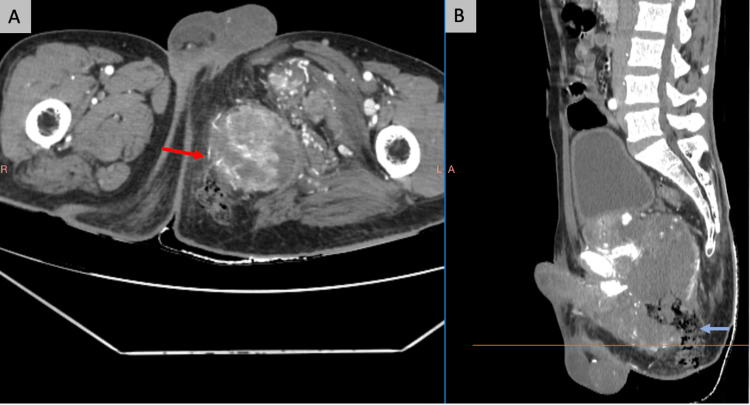
Contrast-enhanced CT of the pelvis in the arterial phase Axial CT image in the arterial phase (A) shows prominent vascular channels extending to the perianal region (red arrow), with the surgical gauze used to control the intraoperative hemorrhage seen adjacent to the vessels and extending into the lesion on the sagittal image (B, blue arrow).

Histopathological examination demonstrated fibroconnective tissue infiltrated by atypical spindle to round pleomorphic cells with hyperchromatic nuclei, suggestive of a high-grade sarcomatous neoplasm (Figure 5). Correlation with the imaging appearance, particularly the characteristic chondroid matrix mineralization and osseous destruction, favored a diagnosis of chondrosarcoma.

**Figure 5:**
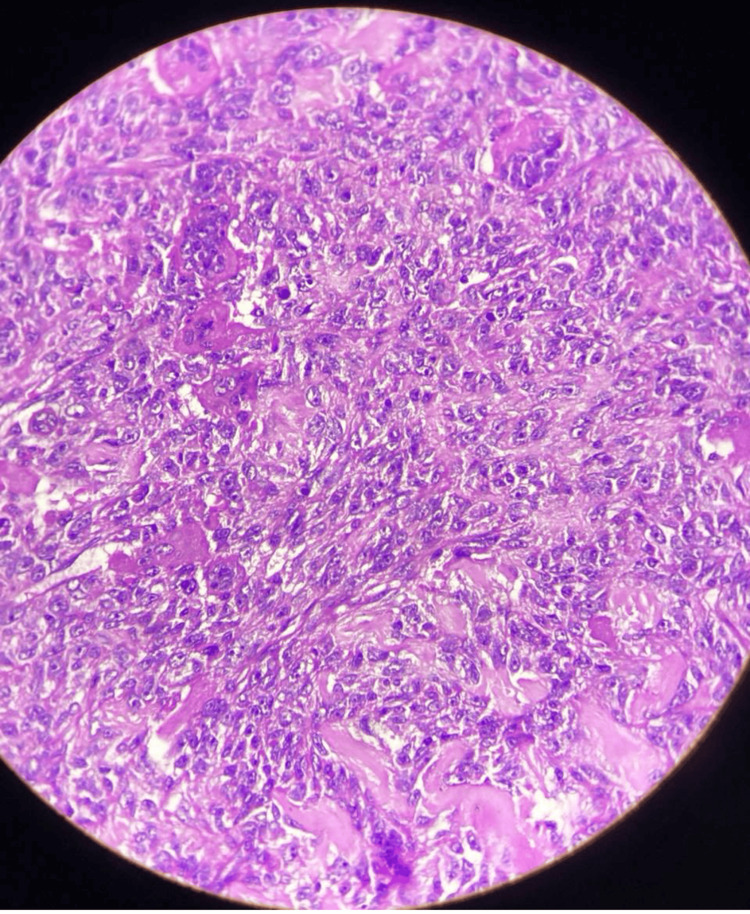
Histopathology slide Histopathological examination demonstrated fibroconnective tissue infiltrated by atypical spindle-to-round pleomorphic cells with hyperchromatic nuclei, suggestive of a high-grade sarcomatous neoplasm.

The patient was subsequently referred to a tertiary care cancer institute for further management. After multidisciplinary evaluation, systemic chemotherapy was advised because of the advanced, locally extensive nature of the tumor and the patient’s poor overall condition, which precluded definitive surgical resection. However, the patient declined further treatment and was discharged against medical advice. He died at home a few days later due to progressive deterioration of his general condition.

## Discussion

Chondrosarcoma represents the third most common primary malignant bone tumor [1] and is frequently diagnosed at a relatively advanced stage because of its slow clinical progression [2]. Several histological variants have been described, including conventional intramedullary, clear cell, juxtacortical, myxoid, mesenchymal, extraskeletal, and dedifferentiated subtypes. Among these, conventional intramedullary chondrosarcoma is the most common and frequently involves the pelvis and long bones, accounting for up to 65% of cases [5].

Radiologically, chondrosarcomas commonly demonstrate characteristic “ring-and-arc” mineralization reflecting enchondral ossification [5]. Other aggressive imaging features include deep endosteal scalloping, cortical destruction, and soft tissue extension [5]. Tumoral calcifications may appear punctate, flocculent, or ring-like in configuration [2]. Once the tumor extends into adjacent soft tissues, the lesion may become clinically palpable because of its large size [2].

In the present case, the imaging findings were highly suggestive of an aggressive chondroid neoplasm. The combination of ring-and-arc matrix calcification, osseous destruction, and extensive soft tissue extension strongly supported the diagnosis. An additional noteworthy feature was the marked internal vascularity seen within the lesion. These abnormal vascular channels likely accounted for the severe hemorrhage encountered during the attempted incision and drainage procedure. Clinically, the deep-seated location of the tumor and the nonspecific presentation contributed to the initial diagnosis of a perianal abscess, particularly in the absence of preoperative imaging.

As these tumors tend to respond poorly to chemotherapy and radiotherapy [1,3,4], wide surgical excision with adequate margins remains the principal treatment modality and provides the best opportunity for local disease control [3].

Although wide surgical excision remains the treatment of choice for localized chondrosarcoma, definitive surgery was not considered feasible because of the tumor’s extensive pelvic involvement, marked vascularity, recent life-threatening hemorrhage, and poor performance status. Following referral to a tertiary care cancer institute, the patient underwent multidisciplinary evaluation, and palliative chemotherapy was advised. However, he declined further treatment and subsequently died at home a few days later because of progressive clinical deterioration. This case illustrates that delayed diagnosis may result in the disease reaching an advanced stage at which curative treatment is no longer possible.

## Conclusions

Pelvic chondrosarcoma may occasionally present in an unusual manner and mimic benign inflammatory conditions such as a perianal abscess. This case highlights the importance of maintaining suspicion for an underlying malignancy in atypical presentations, particularly before surgical intervention, as early imaging may help prevent inappropriate management and potentially life-threatening complications such as severe hemorrhage.

## References

[1] Gelderblom H, Hogendoorn PC, Dijkstra SD, van Rijswijk CS, Krol AD, Taminiau AH, Bovée JV (2008). The clinical approach towards chondrosarcoma. Oncologist.

[2] Ollivier L, Vanel D, Leclère J (2003). Imaging of chondrosarcomas. Cancer Imaging.

[3] Lex JR, Evans S, Stevenson JD, Parry M, Jeys LM, Grimer RJ (2018). Dedifferentiated chondrosarcoma of the pelvis: clinical outcomes and current treatment. Clin Sarcoma Res.

[4] Cao L, Wu Y, Han SM (2021). Dedifferentiated chondrsarcoma: a clinicopathologic analysis of 25 cases. BMC Musculoskelet Disord.

[5] Murphey MD, Walker EA, Wilson AJ, Kransdorf MJ, Temple HT, Gannon FH (2003). From the archives of the AFIP: imaging of primary chondrosarcoma: radiologic-pathologic correlation. Radiographics.

